# Supplementary figures and images for: Loss of Lamin A leads to the nuclear translocation of AGO2 and compromised RNA interference
Source: Nucleic Acids Res. 2024 Jul 12;52(16):9917–35. doi: 10.1093/nar/gkae589 (PMC11381323; doi:10.1093/nar/gkae589)

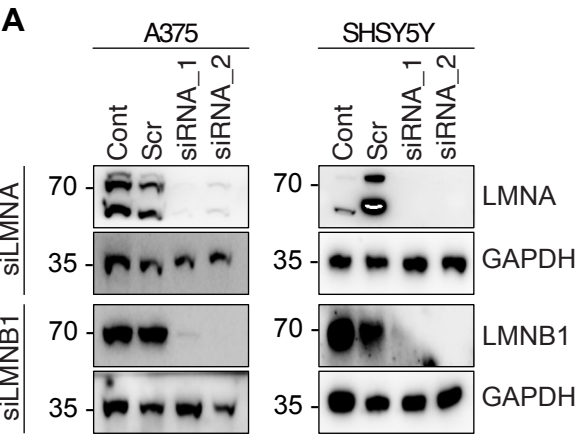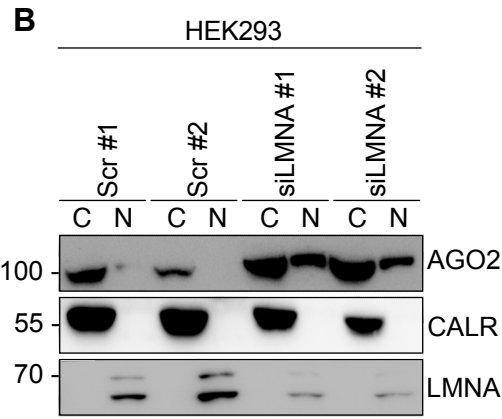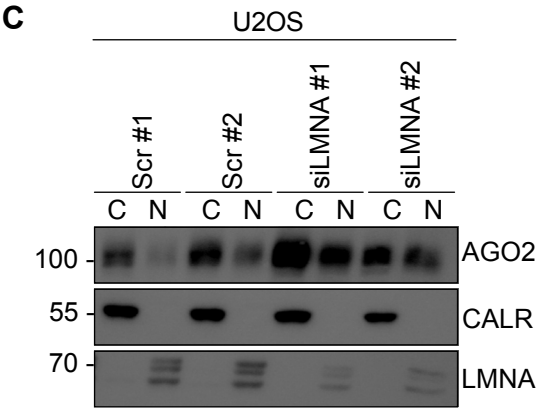

Supplement: gkae589_Supplemental_Files [file gkae589_supplemental_files.zip › Sup Figure 1 related to main Fig 1 IN.pdf]

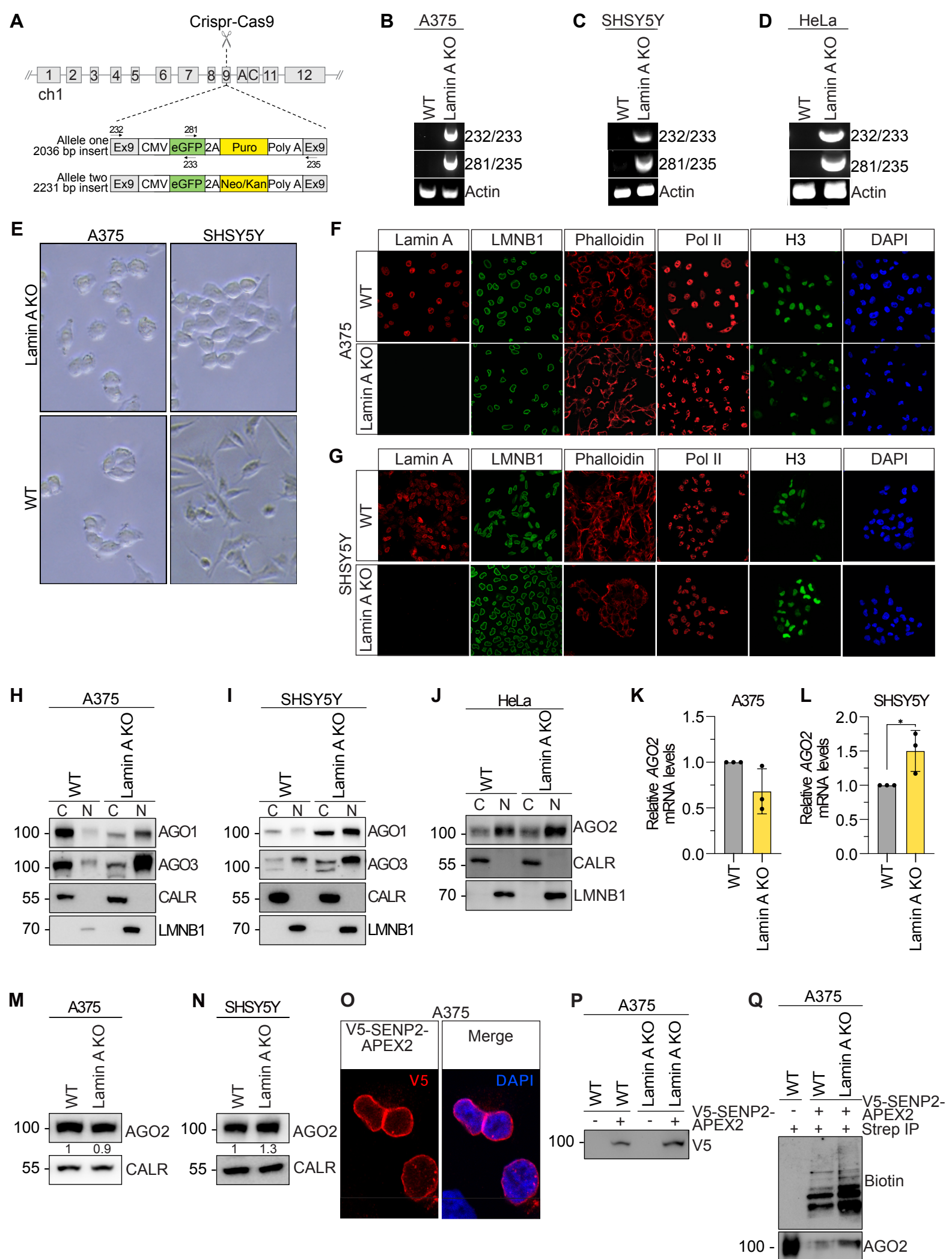

Supplement: gkae589_Supplemental_Files [file gkae589_supplemental_files.zip › Sup Figure 2 related to main Fig 2 IN.pdf]

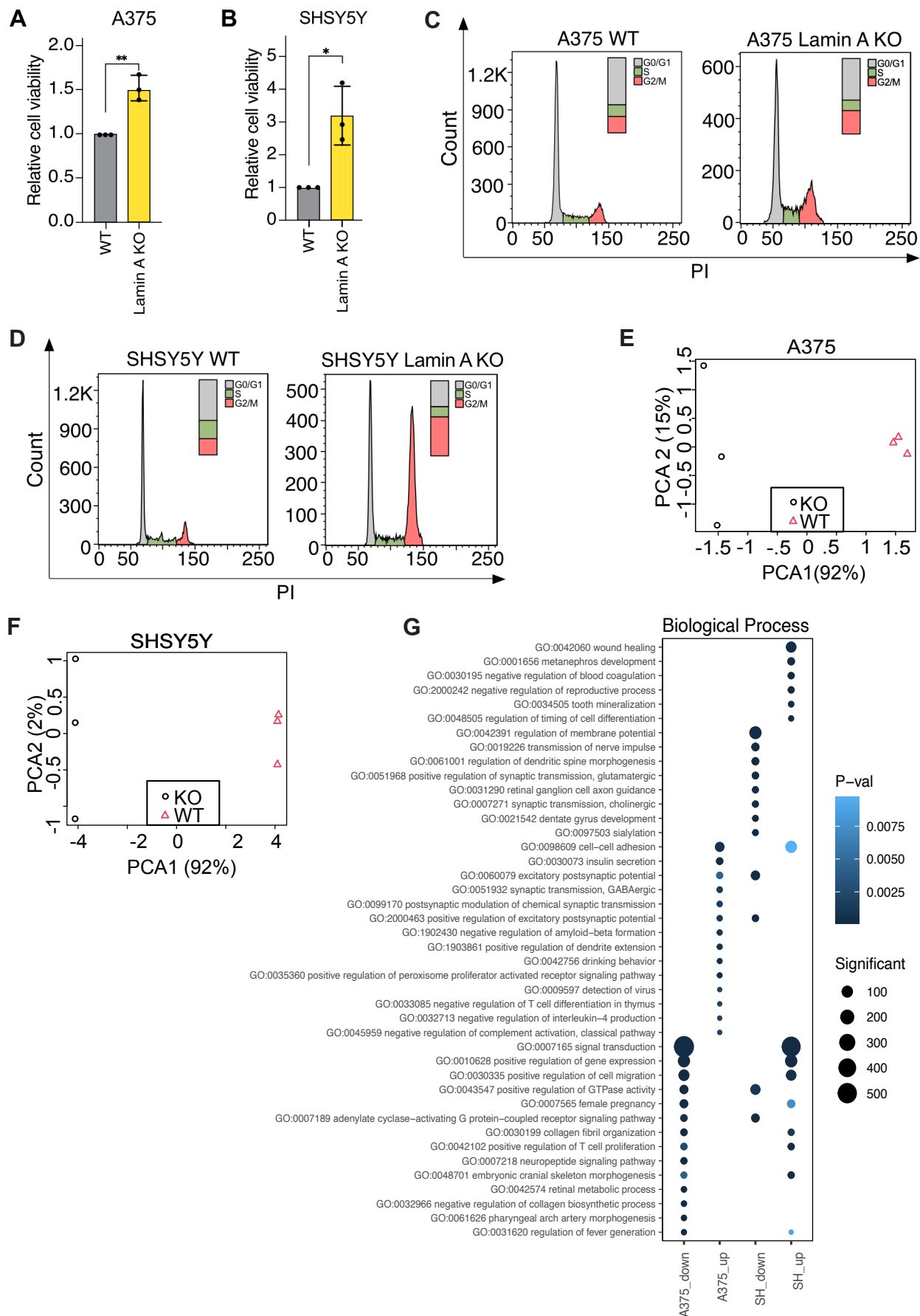

Supplement: gkae589_Supplemental_Files [file gkae589_supplemental_files.zip › Sup Figure 3 related to main Fig 3 IN.pdf]

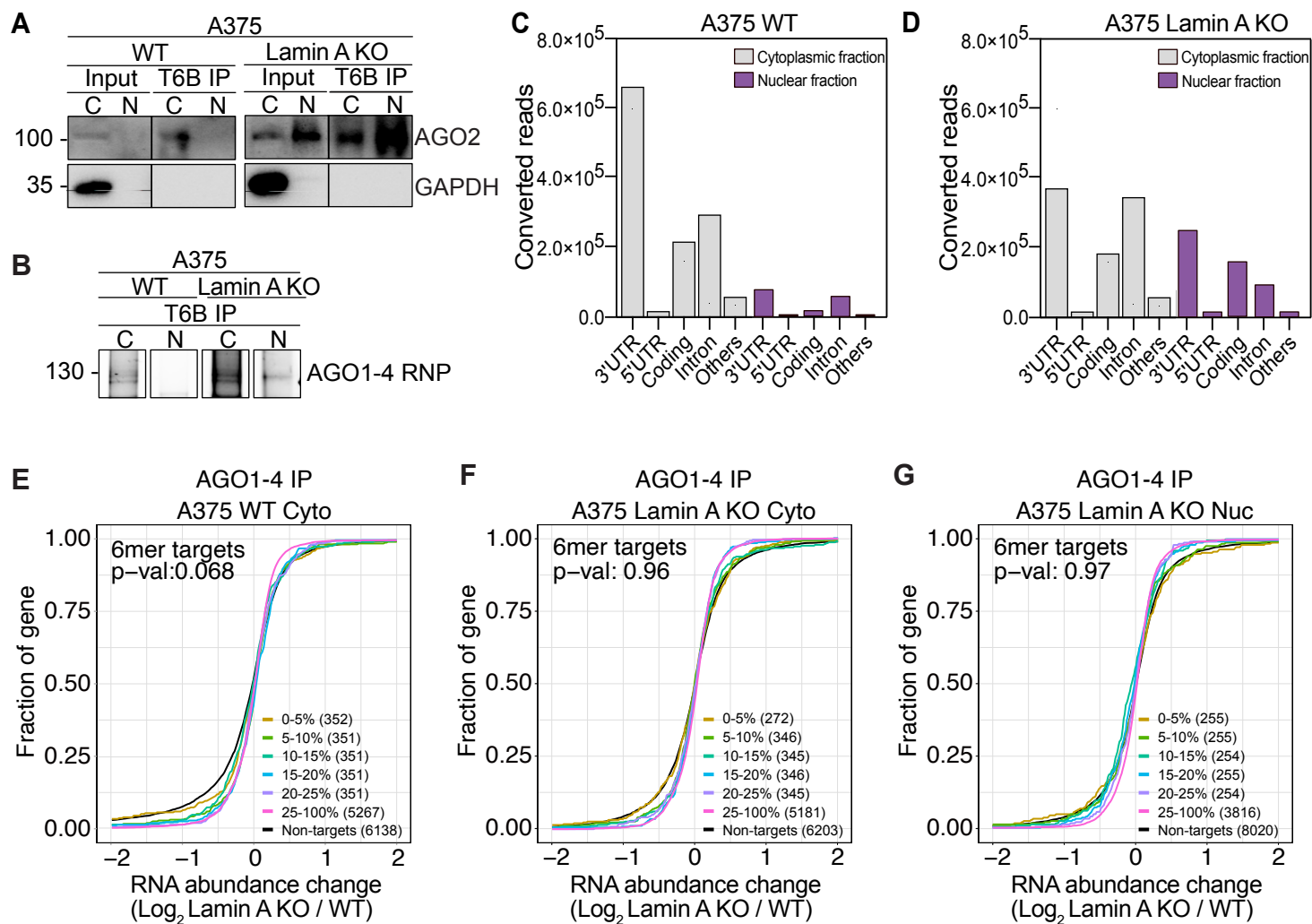

Supplement: gkae589_Supplemental_Files [file gkae589_supplemental_files.zip › Sup Figure 4 related to main Fig 4 IN.pdf]

A

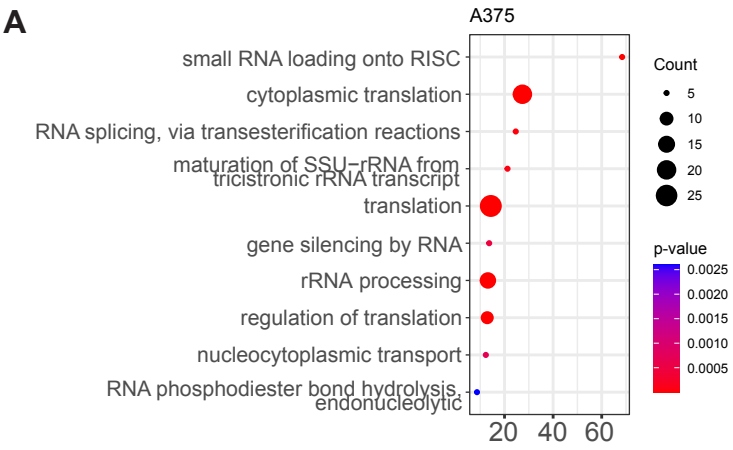

B

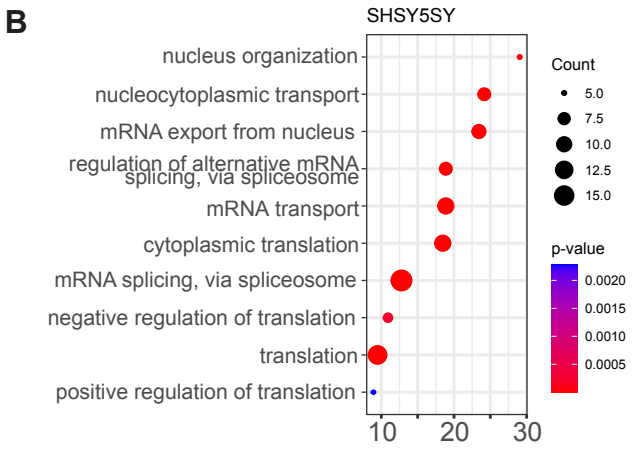

C

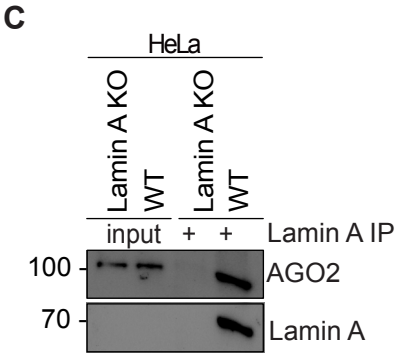

D

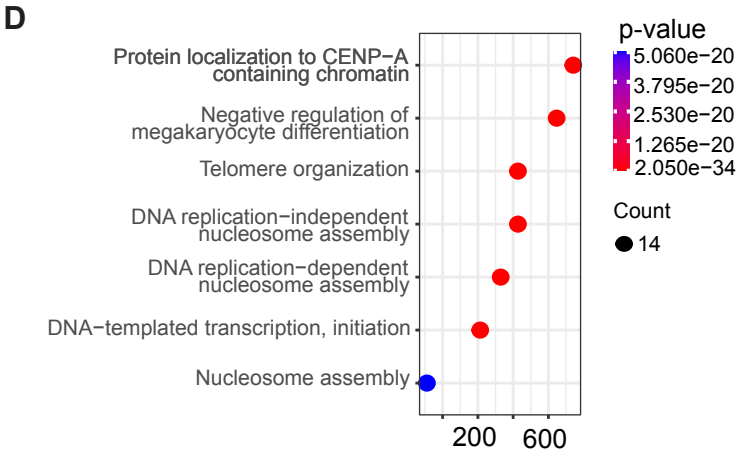

Supplement: gkae589_Supplemental_Files [file gkae589_supplemental_files.zip › Sup Figure 5 related to main Fig 5 IN.pdf]

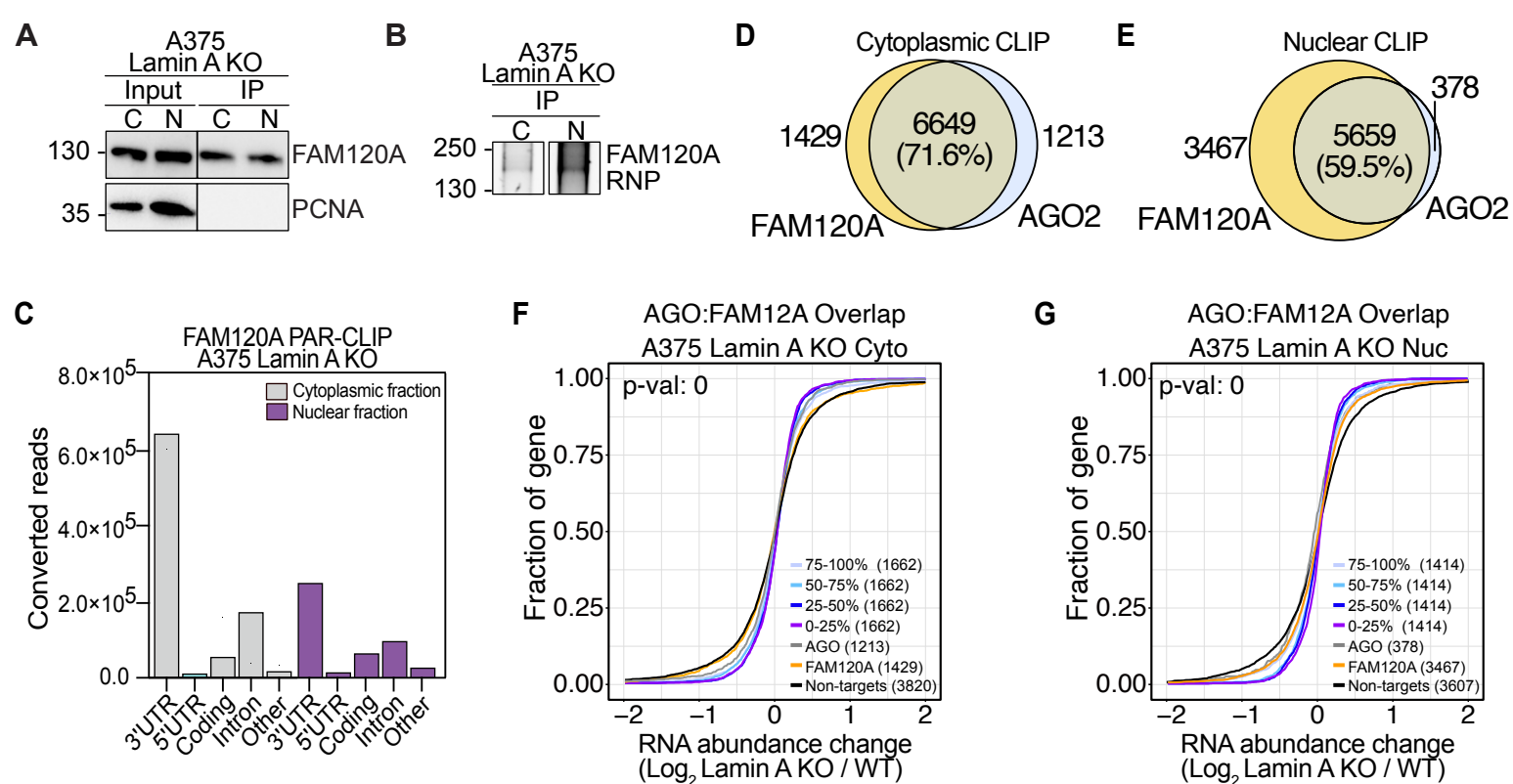

Supplement: gkae589_Supplemental_Files [file gkae589_supplemental_files.zip › Sup Figure 6 related to main Fig 6 IN.pdf]
